# Ross and Joyce Bell as Mentors at the University of Vermont

**DOI:** 10.3897/zookeys.147.2092

**Published:** 2011-11-16

**Authors:** David S. Barrington

**Affiliations:** Pringle Herbarium, Plant Biology Department, University of Vermont

In this contribution I provide an abstract of the unique and long-lasting contribution to the training of students in the natural sciences at the University of Vermont (UVM) that Ross Bell and his wife Joyce made during over 45 years at the University, from 1955 to 2000.

I made a similar contribution to the Bellfest, the symposium celebrating Ross and Joyce’s scientific and educational endeavors, in June of 2010. I have a unique perspective on Ross’s challenges, given our competition to see who can report the worse history of eye disease. Indeed it seems ironic that Ross, who has such a powerful ability as an observer and passion for the beauty of the natural world, should be encumbered by complex visual disorders.

However, it would be wise for a scholarly commentator to identify some shared professional common ground with his subject, and it is in students that we find that common ground. I have had substantial interactions with four of Ross and Joyce’s students: Robert Davidson, Brian Farrell, Jonathan Leonard, and Denise Martin. Their autapomorphies are many (predilections for such diverse things as Lawrence Durrell novels, conga drums, Celtic guitar, and weaving looms), and their synapomorphies are few (capacity for hard work, twisted sense of humor, love of the animals and the science).

In fact, one might argue that I have no perspective on Ross and Joyce’s science, as a comparison of our study organisms reveals. While I study the prudish ferns, their sexual reproduction carried out on separate dedicated organisms hidden and as obscure as possible, Ross and his students have dedicated their lives to the carabids, a group whose sex is manifest and copulation frequent and prominent. Indeed, fern specialists find no particular interest in genitalia, but genitalia are at the center of the coleopterist’s inquiry.

Robert Davidson has kindly provided an anecdote that demonstrates not only the habits of these animals but the predilections of their students. He reports an early visit by our great Quebecois friend André Larochelle, a superb collector of carabids and observer of details of biology, ecology and behavior. At that time, he was still a priest and would show up from time to time in clerical garb. Ross was inside his office in the Marsh Life Sciences Building, and André standing in the doorway half inside and half in the hallway. He had spent most of the spring studying breeding behavior of various beetles and was excitedly sharing some of the stories. But people passing in the hallway saw only a priest announcing in a booming voice, “Yes, we have had MANY copulations this spring!”

In spite of the manifest professional disconnect, I have taken up the task of commenting on Ross and Joyce’s contribution to science and education at UVM, because it seemed appropriate for a pteridologist to speak to Ross and Joyce’s role at UVM: our life’s work addresses equally obscure organisms, asking questions that are equally unfundable.

Ross has an orientation to collection and a scholarly style that yielded a natural-history cabinet and library worthy of the best naturalists of the age of exploration, driven by a deep sense of history. The students attracted to work with him have been susceptible to the same inclination, or as some would have it, affliction. Brian Farrell speaks to this quality: “[Ross’s] welcome, both into the collections, and importantly, into his personal library, convinced me to become an entomologist and an evolutionist. I remember the day I first saw an issue of the journal Evolution in his office, reading it (and the rest of the series) transformed my thinking about what I wanted to do.”

Ross and Joyce’s sabbatical year spent excavating logs in New Guinea captures their enthusiasm for classic natural history and substantial, modern orientation to the science. They spent their time in New Guinea searching for rhysodine beetles: economically worthless, ecologically invisible, and virtually impossible to find. Total yield of animals: 12 beetles from eight dissected logs in three months. Total yield to the science: an ecological and biogeographic synthesis for the rhysodine carabids, centered on New Guinea (Bell 1985). These animals were first included in the carabids by the Bells (Bell and Bell 1962). The early recognition of the rhysodines as a clade of carabids is evidence of Ross and Joyce’s modern phylogenetic perception of evolution. As evidenced by the 1962 paper, the Bells were early advocates and perhaps independently adopted the idea of formal, explicit application of synapomorphy in a parsimony context as the basis for interpretation of evolutionary history.

Ross was at the center of a unique UVM subculture centering on the annual course in field zoology, which each fall brought a proliferation of insect nets out across the campus. The course attracted a unique cadre of students with this model of fascination with the obscure without agenda for fame (though several of his students have failed to remain obscure). Ross’s technique has been simple: as much as possible spend time in the field and the lab with the students, in small groups, obsessed with the animals. It is Ross’s obsession with the animals that most deeply affects his students, and it is the adoption of this passion in their own work that has led to their success and thus his as a mentor. Ross models some key behaviors, imitated by his students, that allow success in this most obscure discipline of ours. Again, Brian Farrell: “His enthusiasm for natural history, his deep insights about evolution, and his warm support of an unusual undergraduate–all were equally important.”

Ross’s sense of humor was probably what did the sorting between the students who would become disciples and students who would flee, thinking Ross was too bizarre. Bob Davidson contributes a story that illustrates this point: “In my second semester ever at UVM, I took Bell’s Zoology 2, an introduction to evolution. Of course I didn‘t know anything about him yet. Over a stretch of two or three weeks, he would talk about various groups of animals, including from time to time large Amazonian fish or reptiles. The first time, it was something that ate this and that, and the occasional bather in the Amazon. A week or so later, another animal that *ate this and that, and the occasional bather in the Amazon*. By the third week, and the third or fourth animal, it was *eats this and that, and the occasional bather in the Amazon*, (pause), *though why the hell anyone would want to bathe in the Amazon after all this is beyond me.*” This tale illustrates both the dry, sardonic nature of Ross’s humor, and the cumulative effect of a joke drawn out over a period of weeks and relying on a student’s memory.

Emblematic of the Bells’ lifelong obsession with these animals is the insect net constantly set in the back of their car to this day, ready at a moment’s notice to capture a new addition to our understanding of the Coleoptera. May there be many more treasures in that net of so many years!
